# *Escherichia coli*, an Intestinal Microorganism, as a Biosensor for Quantification of Amino Acid Bioavailability

**DOI:** 10.3390/s90907038

**Published:** 2009-09-04

**Authors:** Vesela I. Chalova, Sujata A. Sirsat, Corliss A. O’Bryan, Philip G. Crandall, Steven C. Ricke

**Affiliations:** Center for Food Safety-IFSE, and Departments of Food and Poultry Sciences, University of Arkansas, Fayetteville, AR 72704, USA; E-Mails: vchalova@uark.edu (V.C.); ssirsat@uark.edu (S.S.)

**Keywords:** *Escherichia coli*, amino acid bioavailability, microbial biosensors, amino acid/peptide assimilation

## Abstract

In animal diets optimal amino acid quantities and balance among amino acids is of great nutritional importance. Essential amino acid deficiencies have negative impacts on animal physiology, most often expressed in sub-optimal body weight gains. Over supplementation of diets with amino acids is costly and can increase the nitrogen emissions from animals. Although *in vivo* animal assays for quantification of amino acid bioavailability are well established, *Escherichia coli*-based bioassays are viable potential alternatives in terms of accuracy, cost, and time input. *E. coli* inhabits the gastrointestinal tract and although more abundant in colon, a relatively high titer of *E. coli* can also be isolated from the small intestine, where primary absorption of amino acids and peptides occur. After feed proteins are digested, liberated amino acids and small peptides are assimilated by both the small intestine and *E. coli*. The similar pattern of uptake is a necessary prerequisite to establish *E. coli* cells as accurate amino acid biosensors. In fact, amino acid transporters in both intestinal and *E. coli* cells are stereospecific, delivering only the respective biological l-forms. The presence of free amino- and carboxyl groups is critical for amino acid and dipeptide transport in both biological subjects. Di-, tri- and tetrapeptides can enter enterocytes; likewise only di-, tri- and tetrapeptides support *E. coli* growth. These similarities in addition to the well known bacterial genetics make *E. coli* an optimal bioassay microorganism for the assessment of nutritionally available amino acids in feeds.

## 1. Introduction

Biosensors make use of biological components to detect small amounts of chemicals or to characterize samples physiologically or chemically. The advantages of using these biologically based sensors lie in their specificity, sensitivity and portability. Also, unlike chemical or physical analyses, there is no need for large and expensive instrumentation. Biosensors can include enzymes or antibodies, but mainly microorganisms, especially bacteria, have been used. Bacteria have the capacity to grow rapidly in relatively inexpensive media.

The main application for microbial biosensors has been in the environmental area. Whole bacterial cells or sensor bacteria have been used by various research groups for analyzing different compounds, for example, inorganic mercury [1,2], naphthalene [3], and arsenite [4]. Ivask *et al*. [5] reported on a sensor for organic mercury compounds that was constructed using *Escherichia coli*. Recently, the demand for quick and specific analytical tools for food and fermentation analysis has increased and is still expanding. Analyses have been developed for monitoring nutritional parameters, food additives, and food contaminants, among others. A common use for bacterial biosensors is for determination of sugars. Tkak *et al.* [6] used *Gluconobacter oxydans* as a biosensor for detecting total sugars in lignocellulose hydrolysates, and Rotariu *et al.* [7] used yeast cells for selective determination of sucrose. *Escherichia coli* has also been used as a sensor for sugars by Held *et al*. [8], who used it to detect both mono- and disaccharides. Likewise, a number of reports are available on microbial biosensors for specific amino acids such as tyrosine (*Aeromonas phenologenes*), tryptophan (*Ps. fluorescens*) and glutamic acid (*B. subtilis*) [9, 10]. In this review, the focus will be on the application of microbial biosensors for assessing amino acid bioavailability in animal feeds.

The concept of bioavailability is heavily relied on in animal nutrition to recognize the incomplete digestibility and utilization of proteins fed to animals [8]. To compensate the incomplete digestibility of feed proteins and prevent nutritive deficiencies, diets are often supplemented with crystalline essential amino acids. However, if diets are not precisely formulated and amino acids are over-supplemented, excesses are not utilized for muscle development but released by excreta, thus becoming major substrates for ammonia production [9,10]. Ammonia is a colorless gas which is produced as a result of bacterial deamination or reduction of nitrogen substances in manure [11]. An inventory of aerial pollutant emissions emanating from US poultry buildings estimated ammonia emissions to be 664,238 US tons/yr in 2002 [12]. To decrease the level of ammonia emissions in poultry, the usage of diets with reduced and optimized nitrogen content has been recommended [13]. Currently, protein level in feed diets is formulated based on the data for total amino acids. To assist the nutritionist in the preparation of efficient and low-price poultry diets and in the transition from total to bioavailable amino acids application, Applegate *et al.* [14] has suggested that information on both total and bioavailable values of essential amino acids be included on the labels of feed ingredients.

Although rapid and currently in use, chemical methods for the determination of amino acid levels in feed ingredients including high performance liquid chromatography (HPLC) and gas chromatography (GC) are not the most appropriate because they result in higher values than the amounts of amino acids utilized under physiological conditions [15]. Currently, animal assays i.e. *in vivo* methods, are considered standard methods for the determination of bioavailable amino acids because obtained results represent the actual animal response to dietary treatments [16]. These methods however, are very expensive, laborious, and time consuming [17]. The development of alternative methods that are equally reliable but less complex and more rapid is desired. The purpose of this review is to describe and evaluate the theoretical basis of the possible use of *Escherichia coli* as a biosensor for determination of bioavailable amino acids in feed proteins. Major topics include discussion of *E. coli* as a natural inhabitant of the animal alimentary tract, comparison of amino acid/peptide assimilation by the small intestine and *E. coli*, and justification of the potential commercial applications for *E. coli* in the estimation of bioavailable amino acids in protein sources.

## 2. Microbial Biosensors for Amino Acid Quantification

As described by Shockman [18], microbial methods for quantification of biologically available amino acids are accurate, simple, specific and inexpensive. They involve different assay microorganisms (Table 1) and are based on their nutritive requirements for the respective amino acid. An additional requirement is the presence of either externally supplemented or internally expressed proteolytic activities of the assay microorganisms. Most microorganisms do not secrete extracellular proteolytic enzymes and, therefore, can be used for the assessment of only enzymatically pre-treated protein sources. Stott and Smith [19] and Bos *et al.* [20] used the protozoon *Tetrahymena pyriformis* in a protein quality evaluation of test materials mainly because of its proteolytic capabilities. It requires lysine to grow and therefore can be used for its quantification. However, a disadvantage of this microorganism is the relatively long incubation period (approximately four days). In addition, laborious and tedious cell count is the preferred method to measure the growth response to lysine. Other microorganisms such as *Streptococcus faecalis*, *S. zymogenes*, *Leuconostoc mesenteroides*, *Pediococcus acidilactici*, and *Lactobacillus arabinosus* have also been used in the determination of bioavailable amino acids (Table 1) [21–24]. Growth responses of these bacteria to the corresponding amino acids were measured by either optical density (OD) or titration of the produced lactic acid. The quantification of the latter was performed in the stationary phase of the microbial growth, taking approximately 72 h. Since other organic acids are also produced, the accuracy of the assay is influenced by whether total or lactic acid production is measured. In addition, lactic acid bacteria are characterized with relatively complex nutritive requirements which include vitamins, purine, and pyrimidine media supplementation to ensure optimal growth [18,25].

The most highly investigated microorganism for amino acid bioavailability quantification is *Escherichia coli* (Table 2). Although not naturally auxotrophic for amino acids like the lactic acid bacteria, *E. coli* has multiple advantages over other assay microorganisms [26]. *E. coli* is characterized with the lowest doubling time among other bacteria [27]. It is easy to grow and has simple media requirements. *E. coli* genetics is well established and can be easily manipulated to generate mutants with desired characteristics. Furthermore, *E. coli* is a part of the intestinal microflora of most animals and humans with presumably similar assimilation of amino acids and peptides which is a necessary prerequisite for the bacterium to serve as a biosensor microorganism for these compounds.

## 3. *E. coli* as a Gut Microorganism

The microflora of the gastrointestinal tract (GI) is diverse and complex. According to Moore and Holdeman [28], more than 400 bacterial species have been found in the colon. Gastrointestinal microflora play an important role in the determination of the total physical condition of animal species. The incredible variation in microbial population is determined by host physiology, environmental conditions, and microbial interactions. Mostly anaerobic but also aerobic as well as facultative anaerobic microorganisms have been isolated and characterized [29–31]. *E. coli* is a persistent inhabitant in the alimentary tract of both healthy and sick mammals [32,33] and can reach a level as high as 10^10^ bacterial cells per gram of fecal sample of an adult animal [34]. As a facultative anaerobe it is capable of growing with minimal oxygen levels. This feature combined with its ability to survive over a relatively large range of pH makes it abundant even under conditions unfavorable for bacterial growth [35].

The overall population level of bacterial cells and in this particular case of those of *E. coli* along the GI tract is highly dependent on location. The human stomach is generally considered nearly sterile because of the low gastric pH [36]. The small intestine is a transition zone between the stomach and the large intestine and contains a highly diverse and dense microbial population. The upper part of the small intestine is characterized by very low microbial counts with nearly complete absence of coliforms. In the distal ileum, gram negative bacteria outnumber gram positive and the coliform titer reaches 10^7^ colony forming units (cfu)/mL. The reason *E. coli* does not inhabit the upper part of the small intestine may be due to an inability to adhere to the epithelium of the proximal tract and thus it is washed out at a rate that does not allow for persistent development in this zone [34]. However, some pathogenic strains can adhere to the intestine because of their ability to produce lectins. For example, human enteropathogenic *E. coli* strains are found to bind to the human intestine. Puzstai *et al.* [37] observed adhesion of *E. coli* RDEC-1 to rabbit intestine.

The colonization of the intestine by *E. coli* strains producing K99 and F41 lectins is considered a major cause of neonatal diarrhea in calves [38]. Interestingly, plant lectins found in diets, although not of bacterial origin, are also found to promote *E. coli* proliferation in the small intestine. According to Wilson *et al.* [39], coliform numbers increased from an initial count of approximately 10^3^ cfu/g in small intestinal rat tissue to 10^10^ to 10^11^ within the first 24 to 48 hours of feeding a kidney bean diet. Although the upper part of the small intestine in healthy individuals is generally considered nearly free of coliforms, some studies from India [40] and South America [41] suggest the presence of enterobacteriaceae including *E. coli* and *Klebsiella pneumonia* in the upper intestines in healthy humans.

The large intestine is the part of the GI tract most heavily inhabited by microorganisms [42]. While the abundance of *E. coli* in the colon is mainly associated with pathogenic consequences [43], this bacterium was found to be important in maintaining the diversity of indigenous microflora as well [44]. By utilizing the oxygen diffused into the lumen, *E. coli* enables sensitive anaerobic bacteria to survive and participate in fermentative processes which are considered important for human health [29].

## 4. Protein Digestion in Animals. Small Intestine as the Primary Site for Amino Acid and Peptide Uptake

Proteins are major components of animals. The protein content of mature animals accounts for 15 to 18% of the body weight [45]. The building blocks of proteins are amino acids that are either newly synthesized or derived from hydrolyzed food proteins. Protein digestion starts with the production of oligopeptides (containing more than three amino acids) in the animal stomach as a result of the direct exposure to pepsin which attacks peptide bonds involving aromatic amino acids. Oligopeptides are further hydrolyzed by pancreatic proteases along the digestive tract. Pancreatic proteases are divided into two major subgroups consisting of endopeptidases and exopeptidases. Endopeptidases including trypsin, chymotrypsin, and elastase are secreted into the duodenum as inactive precursors [46]. After being transformed into active forms, they catalyze the disruption of endopeptide bonds thus releasing relatively large oligopeptides. External peptide bonds of polypeptides and proteins are digested by pancreatic exopeptidases. The complex hydrolysis action of pancreatic enzymes on feed proteins results in a mixture of free amino acids, di- and tripeptides and large peptides. The large peptides cannot be assimilated by the small intestine and must be further hydrolyzed by brush border-membrane peptidases [47]. According to Johnson [50], luminal digestion of protein meal produces approximately 40% amino acids and 60% small peptides. The small intestine is the primary place where amino acids and small peptide absorption occurs although the absorptive capacity along the length of small intestine varies [48].

As an inhabitant of the small intestine without the capability to secrete extracellular proteases, *E. coli* is forced to assimilate the mixture of amino acids and peptides derived from the enzymatic degradation of food proteins [49,50]. This may lead to a competition between *E. coli* and the small intestine for protein digestive products as an eventual overgrowth of even non-pathogenic strains may slightly reduce the amount of nutrients available for the animal [37]. Interestingly, although genetic and physiological differences between the prokaryotic *E. coli* and eukaryotic intestinal epithelial cells are considerable, the assimilation of the same compounds implies probable similarities in the mechanism of their absorption which is a must if *E. coli* is to be used as biosensor for bioavailable amino acid quantification [51,52].

## 5. Amino Acid and Peptide Transport in Small Intestine

Amino acids can enter the cells by simple diffusion, facilitated diffusion (Na^+^- independent), and active transport (Na^+^- dependent) [48,53]. The contribution of each transport system to the total amount of amino acids entering the cell is highly dependent on substrate concentration [48]. The active transport is energetically expensive for cells. An amino acid is co-transported with a Na^+^ into the cell against the amino acid concentration gradient which requires additional energy input. It is provided by a membrane Na^+^ gradient which in turn is generated by a Na^+^/ K^+^-ATP dependent pump retaining low intracellular and high extracellular concentrations of Na^+^ [48]. Because of the high energy expense, the active transportation of amino acids, in general, occurs at low extracellular concentrations. Stevens *et al.* [54] observed that phenylalanine enters the cells by active transport only at concentrations below 1 mmol/L. When leucine uptake was studied, it was reported that the combined active and facilitated diffusion contributed to leucine transport primarily at concentrations no higher than 5 mmol/L [55]. For lysine and methionine, the active mechanism of transport occurred at an even lower concentration, namely 1.0 mmol/L [56]. As the substrate concentration increases the contribution of facilitated transport and passive diffusion to the total amount amino acid uptake increases, implying simultaneous presence of several mechanisms for amino acid uptake. Most amino acids are transported by more than one transporter which provides not only high energy efficiency but also backup capacity for absorption in the case of mutational inactivation [57]. Smith *et al.* [58] confirmed the presence of high-affinity and low-affinity carrier systems for serine, alanine, and methionine transport into rabbit ileal enterocytes. B^o^-type Na^+^-dependent and B^o^-type Na^+^-independent transporter systems for neutral amino acids have been characterized at the molecular level and reported to have a concentration-based mode of regulation [59–61].

The precise number of amino acid transport carriers is not known. Christensen [62] described 12 distinct amino acid transport systems. Stevens *et al.* [54] characterized and added three more intestinal amino acid carriers to the list. Although they differ in their location and kinetic characteristics, all of them have a common requirement for the possession of amino- (or imino-) and carboxyl groups by the substrate being transported [63]. Based on this, Munck [64] concluded that intestinal carriers for amino acids can transport only amino acids.

Different transport systems exist for the import of small peptides. By studying intestinal amino acid and peptide transport in the rabbit ileum, Rubino *et al.* [65] established that free amino acids do not affect the transport of dipeptides and vice versa. Amino acid and peptide competition experiments have been used to confirm the existence of independent transport systems for amino acids and small peptides in intestines [52]. The independent functions of amino acids and peptide carrier systems have been further demonstrated by studies performed with patients with deficiencies in amino acid metabolism such as Hartnup disease and cystinuria [66].

Since the Na^+^-dependent amino acid transport system was well understood, it was intuitively assumed that the same mechanism for peptide cell entry also occurred. However, by using brush-border membrane vesicles, Ganapathy *et al.* [67] demonstrated that peptide transport did not depend on Na^+^ gradient. Coupling of intracellular peptide concentration and H^+^ gradient was reported [68]. Peptide transport in the small intestine driven by proton motive force was also confirmed by Miyamoto *et al.* [69]. However, when dipeptide transport was studied in rabbit, rat, and human intestinal brush-border membrane vesicles (BBMV), Rejendran *et al.* [70] noted that generated proton motive force could not drive the accumulation of dipeptides against their concentration. Similar observations were reported by Wilson *et al.* [71] who studied tripeptide transport in human jejunal BBMV. In addition, the transport of glutathione in rabbit BBMV appeared to be energized by mono- and divalent (Ca^2+^) cations but not by H^+^ [72]. Based on these conflicting data, Webb *et al.* [73] concluded that two classes of carrier-mediated peptide transporters exist: one that is energized by proton motive force, and another that is energized by cations other than H^+^ and Na^+^.

Regardless of the mechanisms for the entry of peptides into the cells however, only di- and tri-peptides can be transported by carriers [48]. One of the methods most widely used to determine the maximal number of amino acid residues for peptide absorption was based on peptide disappearance from the gut lumen in the presence of specific inhibitors of brush border peptide hydrolases [74]. Using such a method, Adibi [75] concluded that only di- and tri-peptides are actively transported by carriers in the small intestine with very low probability for tetrapeptides to actively cross the cell membrane. Instead, they are hydrolyzed to free amino acids and smaller peptides by brush border peptidases [47].

Except for the number of amino acid residues in peptides, the presence of amino and carboxyl terminal groups appeared to be critical for peptide uptake. Addison *et al.* [76] observed that the blocking of N-terminal group by acetylation or methylation or the C-terminal group by esterification highly decreased the peptide affinity for transporters. Cyclization of dipeptides was a different approach to evaluate the importance of terminal amino- and carboxyl groups [77]. Subsequent experiments demonstrated that cyclic dipeptides failed to compete with the corresponding linear dipeptides as evidenced by their substrate affinity constants.

Stereospecificity of dipeptide transporters has also been studied as an important characteristic. Asatoor *et al.* [78] observed that the absorption rate of l-Ala-l-Phe and l-Leu-l-Leu is much greater than those of their respective d-d isomers. A thorough investigation on the stereospecifity of kidney oligopeptide/H+ symporter was performed by Daniel *et al.* [77]. The research group established 60-fold reduction in the transporter affinity when l-Ala in Ala-Gly dipeptide was substituted by its d-isomer. The replacement of –COOH terminal residue with its d-isomer led to even greater reduction in the affinity. The substitution of both l-amino acids with their d-forms completely abolished the uptake of the dipeptide. Although they observed less reduction in the substrate affinity of lipophilic peptides with d-amino acid substitutes, the overall conclusion was that the peptide stereospecifity was an important determinant of transporter affinity. Since the biologically active form of amino acids is the l-form and only l-amino acids are utilized for the synthesis of new proteins, these observations were not surprising [79].

## 6. Comparative Characteristics of Amino Acid and Peptide Transport in *E. coli*

The overall comparison of amino acid and peptide transport in *E. coli* and animal small intestine is outlined in Table 3. *E. coli* can synthesize all of the amino acids from inorganic compounds and glucose but it is also capable of transporting intact amino acids into the cell from the extracellular environment. For *E. coli*, the amino acids can serve as a sole carbon or nitrogen source. However, the catabolism of amino acids is energetically expensive and in a natural environment such as the small intestine, where other carbon sources are also available, the uptake of amino acids is designed to replenish the intracellular pool of amino acids necessary for the biosynthesis of proteins, nitrogen transfer, or osmotic protection [50,80,81].

As in the small intestinal epithelial cells, there are three ways for amino acids to enter the bacterial cells: passive diffusion, facilitated diffusion and active transport [82]. Passive diffusion and facilitated diffusion do not require additional energy input as they occur at relatively high concentrations of substrates. However, active transporters are activated at low concentrations of substrates with a Michaelis-Menten (Km) constant varying in the range of 1 to 10 μM [83]. Some high affinity transporters such as for l-glutamine and l-proline were reported to have even lower Km, namely 0.1 and 0.7 μM respectively [84].

Just as with intestinal transporters, microbial active uptake systems require energy input since substrates are transported against their concentration gradient. Common energy sources for the active transport in bacterial cells are H^+^- and Na^+^ -membrane concentration gradients, and ATP hydrolysis. While in the small intestine active transport of amino acids is dependent mostly on the Na^+^-membrane gradient [47], in *E. coli*, all three energy sources can be used depending on transporters and amino acid (s) being transported. The specific tyrosine and leucine transporters are energized by H^+^-membrane gradients [85], while the ABC transporters for aromatic amino acids and hystidine are energized by ATP hydrolysis [86]. Although the Na^+^-membrane gradient is not considered typical for prokaryotes, the transport of L-serine and L-threonine [87] as well as those of proline [50] is considered Na^+^-dependent.

Based on kinetic analysis, several distinct transport systems for amino acids with very little overlap have been described in *E. coli* [83]. Distinct transport systems exist for branched amino acids (isoleucine, leucine and valine) and aromatic amino acids (tyrosine, phenylalanine and tryptophan). Distinguishable transport systems for hystidine and proline have also been described. In addition, multiple transporters may exist for the amino acids belonging to one group [85, 88]. They differ mainly in their affinity to the substrates and are regulated by the intracellular level of the respective compound. For example, the low affinity transport system (LIV-II) for the branched amino acids usually operates under optimal concentrations of the respective substrates, while the high affinity carrier is activated by leucine starvation. A third system, specific only for the transport of leucine, also exists and is considered a component of the branched chain amino acid family transport system [85]. There are two systems for lysine, ornithine, and arginine [89] as well as for aspartate and glutamine [50]. Five transporters for the intracellular accumulation of aromatic amino acids have been identified and studied [90]. In short, the diversity in the transport systems for amino acids in *E. coli* is much higher than the uptake systems in the small intestine of the host. The reason for this may relate to the requirements for bacterial populations to meet their physiological needs under different environmental conditions that are unfavorable for their growth. Regardless of these differences, a common feature of amino acid transport in both small intestine and *E. coli* is the transport of amino acids by active mechanisms at low substrate concentrations and the stereospecifity of transporters. As Piperno and Oxender [83] stated, all amino acid transporters except for those of alanine, glycine and serine, are highly stereospecific, being able to transport only l-amino acids.

*E. coli* absorb not only free amino acids but also small peptides. As in the small intestine, there are distinct transport systems for the import of amino acids and peptides [82,91]. The isolation of mutants deficient in transport system in either of them as well as the experiments involving competitive inhibition between amino acids and dipeptides unambiguously proved the independent existence of amino acid and dipeptide transport [92]. *E. coli* dipeptide transport resembles that of the small intestine in its specific requirement for N- and C-terminal groups and the blockage of these functional groups prevents dipeptide uptake [91]. Like intestinal transporters, *E. coli* dipeptide transport systems are also highly stereospecific, the reason potentially being the inability of *E. coli* to convert d-stereoisomers into utilizable l-forms [92].

The difference in the peptide utilization by small intestine and *E. coli* cells is the existence of a distinct oligopeptide system. The *oppABCDF* system is located at 27 min of the *E. coli* genome and is able to transport peptides containing from two to five amino acid residues [93,94]. However, it appeared that the Opp was specifically capable of transporting and recycling exclusively cell wall peptides which may explain the existence of this transporter in *E. coli* but not in intestinal cells [95]. These findings agreed with previous observations that some oligopeptides, although satisfying the structural requirements for oligopeptide transport, were unable to enter *E. coli* cells [96]. While tetrapeptides of lysine and arginine poorly supported *E. coli* growth, pentapeptides and higher homologues completely abolished the bacterial propagation [96].

Consistent with other active transports, peptide transport in *E. coli* requires energy. While in the small intestine proton motive force is considered a primary energy source, in *E. coli* cells this role is assigned to ATP hydrolysis [97–99]. ATP hydrolysis occurs concomitantly with the function of periplasmic binding protein-dependent transport systems including Opp which are considered to play a major role in peptide transport in *E. coli* [100–102]. By studying the transport of Opp-specific substrate l-prolylglycylglycine, Mimmack *et al.* [102] recorded ATP consumption in the wild type strain but not in the *opp^−^* mutant. In addition, proton motive force (pmf) was discovered to take part in peptide transport as well [103]. It seems however, that pmf-dependent systems were specific only for the transport of di- and tripeptides [101].

Although *E. coli* and intestinal cells are structurally very different, it appears that substantial similarities in amino acid and peptide uptake between both biological subjects exist. In fact, amino acid transporters in both intestinal and *E. coli* cells are stereospecific, delivering only the respective biological l-forms. The presence of free amino- and carboxyl groups is critical for amino acid and dipeptide transport. Di-, tri- and tetrapeptides can enter enterocytes; only di-, tri- and tetrapeptides support *E. coli* growth. These similarities along with other specific characteristics are very important prerequisites for a test microorganism to achieve an optimal correlation between potential microbiological assays and the corresponding animal assays currently in use for amino acid bioavailability quantification.

## 7. *E. coli* in Use as a Biosensor to Assess Amino Acid Bioavailability in Feed Proteins

Animal assays, i.e. *in vivo* methods, are considered standard methods for the determination of bioavailable amino acids because the results obtained represent the actual animal response to dietary treatments [16]. Although accurate and reliable, they are laborious, time consuming, and expensive to be used on a daily basis. In contrast, microbiological assays do not require elaborate instrumentation. They are simple, specific and inexpensive. To be used as a test microorganism, a bacterium should be an auxotroph i.e. it should be deficient for the amino acid of interest. The cell growth of such an auxotroph is a direct function of the concentration of the compound being assayed [104]. Therefore, the amount of the amino acid in the medium can be determined by measuring bacterial cell growth. Wild type *E. coli* can synthesize all 20 amino acids growing in medium containing only a carbon source and inorganic salts. Consequently, the wild type *E. coli* cannot be used directly for amino acid quantification. Although not a natural auxotroph for amino acids, *E. coli* has multiple advantages over other assay microorganisms. It is easy to grow and it does not require elaborate equipment and expensive nutritive ingredients. In addition, *E. coli* is characterized with the lowest doubling time among other bacteria which determines the relatively short time (7 to 8 h) required to reach stationary phase [105]. *E. coli* genetics is well established and can be easily manipulated. Multiple mutants have been created and studied for the purpose of bioavailable amino acid quantification (Table 2).

The extensive research towards the development of a stable lysine auxotroph strain is determined by the limiting amount of this amino acid in feed ingredients and the high variability of its bioavailability [106]. The accurate measurement of bioavailable lysine in feed ingredients would prevent the addition of lysine in excess as a “safety factor” used in the animal practice to compensate the uncertainty about lysine bioavailability and the animal requirement for this nutrient [107]. The diversity of the detection modes used in these types of assays is also presented in Table 2. With the discovery and the application of green fluorescent protein (GFP) as a reporter [108,109], the potential of *E. coli* assays for rapid and accurate estimation of bioavailable lysine even under non-sterile conditions increased [110]. Finally, as a gastrointestinal microorganism competing with intestinal cells for some dietary derived amino acids and peptides available in the animal gut, *E. coli* appear to be a promising alternative to test animals used in the quantification of bioavailable amino acids.

The idea of using *E. coli* to assay accurately bioavailable amino acids in proteins was suggested by Gilvarg and Katchalski [96] and later by Payne [91], who showed that there were no differences in *E. coli* lysine auxotroph growth responses to either free lysine or lysine in peptide linkage of di- and tripeptides. This observation enabled bioavailable lysine (free lysine, di- and tri- lysine containing peptides) to be evaluated simply by the construction of a crystalline lysine standard curve. Since *E. coli* does not naturally excrete extracellular proteases and peptidases, an enzymatic pre-digestion of a protein is required for an *in vitro* analysis. Therefore, the successful use of *E. coli* to assay bioavailable amino acids in feed proteins was dependent on the development of a pre-digestion procedure that closely simulated the *in vivo* protein digestion occurring in the gastrointestinal tract. A variety of single enzymes and enzyme combinations have been explored to degrade feed proteins to an amino acid/peptide mixture that mimics the *in vivo* digestive protein product. Payne *et al.* [49] reported that among pepsin, trypsin, chymotrypsin, pronase and peptidase, the combination of pronase and peptidase was the best for the estimation of bioavailable lysine in proteins when using an *E. coli* lysine auxotroph. By exploring the digestive profiles of the studied protein sources, they established that the combination of pronase and peptidase released only negligible amounts of oligopeptides larger than pentalysine which was an important prerequisite due to the size requirements for the digestive products. Protein cleavage products should be small enough to pass the bacterial cell wall and reach their cytoplasmic carriers if *E. coli* is to be used as a test microorganism [49,111]. When Erickson *et al.* [112] used both pronase and peptidase to pre-digest animal protein feeds, they obtained a correlation of 0.94 between lysine bioavailability determined by using *E. coli* lysine auxotrophs and previously published chick bioassay data. Following the same procedure but measuring the fluorescence emission of an *E. coli* lysine auxotroph transformed with a *gfp*mut3, Chalova *et al.* [113,114] were able to determine bioavailable lysine in feed proteins including sorghum, soybean meal, cotton seed meal, meat and bone meal, chick starter and finisher, and swine starter in a 6-hour period. No significant differences in estimated bioavailable lysine were observed when data were compared to the data for bioavailable lysine generated by chick bioassay on the same feed samples except for sorghum.

An *E. coli* methionine auxotroph was used to quantify bioavailable methionine in protein sources [115]. The strain was transformed with a plasmid carrying a gene for a green fluorescent protein. Bacterial growth response to the protein derived methionine was measured either turbidimetrically or by fluorescence. The method was also adapted for application using microtiter plates which not only decreased the time required for an assay completion if compared to tube-based assays but also diminished material and equipment expenses [116]. Microbial *E. coli* based assays for determination of bioavailable amino acids in protein sources performed in microtiter plates highly reduced the volume of media and incubation space required and enabled simultaneous quantification of a large number of samples.

Methionine and lysine bioavailability in foods were also quantified by Hitchins *et al.* [117]. Their method was based on the study of Tuffnell *et al.* [118] who observed that the extent of the synthesis of β-galactosidase strictly depends on the externally supplemented lysine. Following the same procedure, Hitchins *et al.* [117] determined the bioavailability of cysteine, threonine and tryptophan by measuring the activity of β-galactosidase in 17 pronase digested foods. They concluded that the method was reliable and accurate enough to predict the first and second limiting amino acid.

In conclusion, *E. coli* can be considered a promising test microorganism for the evaluation of bioavailable amino acids in protein sources. The intrinsic properties of these bacteria as well as the numerous similarities between protein digestion product assimilation by *E. coli* and the small intestine make the development of accurate and reliable microbial methods for bioavailable amino acid quantification possible. The time required for the completion of the microbial assay (6–8 hours) [110,119] is much less than the time required for the chick bioassay (two to three weeks) [17,113,120]. Based on the current results published in literature, the microbial assay appears to have other advantages as well. Factors such as low metabolizable energy and low amino acid content of feed samples do not affect *E. coli* assays while they are of serious consideration if an animal assay is to be used for those sample analyses [120]. Environmental temperature, sex, age and species highly influence the animal response to the amino acids as well [121]. In addition, animal methods are not suitable for the generation of high through-put volumes of data because only one amino acid can be measured at a time. However, *E. coli* auxotrophs for different amino acids could be generated and corresponding biosensors developed for the estimation of a large set of nutritionally important amino acids. The different strains could be applied simultaneously using microtiter plates and the bioavailability of all essential amino acids in protein sample(s) determined.

## Figures and Tables

**Table 1.** Assay microorganisms for amino acid quantification. OD refers to optical density.

**Assay microorganism**	**Amino acid assayed**	**Assay response**	**Detection method**	**Reference**
*Streptococcus faecalis*	Valine, Leucine Threonine	Cell growth Acid production	OD Titration	Blackmore and Parry [22] Cardinal and Hedrick [21]
*Streptococcus zymogenes*	Tryptophan, Methionine	Cell growth	OD	Wells *et al*. [23]
*Leuconostoc mesenteroides*	Methionine Lysine, Arginine, Proline, Phehylalanine, Methionine, Cystine, Serine, Alanine, Aspartic acid	Cell growth Acid production	OD Titration	Blackmore and Parry [22] Cardinal and Hedrick [21]
*Lactobacillus arabinosus*	Leucine, Isoleucine, Valine, Glutamic acid	Acid production	Titration	Cardinal and Hedrick [21]
*Pediococcus acidilactici*	Lysine, Methionine	Cell growth Acid production	OD Titration	Odunfa *et al*. [24]
*Tetrahymena pyriformis*	Lysine	Cell growth	Cell count	Stott and Smith [19]; Bos *et al*. [20]
*Escherichia coli*	Cysteine, Glutamine, Methionine, Lysine, Threonine, Tryptophan	Variable	Variable	Table 2 this review

**Table 2.** *E. coli* strains for bioavailable amino acid quantification. OD refers optical density, GFP denotes green fluorescent protein.

***E. coli* strain**	**Amino acid analyzed**	**Assay response**	**Detection type**	**Reference**
*E. coli* DM 800	l - Cysteine	β-galactosidase	β-galactosidase activity	Hitchins *et al.* [117]
*E. coli* M 5004 (trpA^−^ gln^−^)	l - Glutamine	Cell lyses	OD	Krapf and Bode [115]
*E. coli* ATCC 23798 (thi^−^)	l – Methionine	Cell growth	OD Fluorescence	Froelich *et al.* [115]
*E. coli* M-26-26	l – Lysine	Cell growth	OD	Payne *et al.* [49]
*E. coli* M 2626	l – Lysine	β-galactosidase	β-galactosidase activity	Tuffnell and Payne [118]
*E. coli* CBK 140 (*lysA*::Tn5)	l – Lysine	β-galactosidase	β-galactosidase activity	Hitchins *et al.* [117]
*E. coli* ATCC 23812 (lys^−^)	l – Lysine	Cell growth	Bioluminescence	Erickson *et al.* [123]
*E. coli* Δ*lysA* (*lysA*^−^)	l – Lysine	Cell growth	OD	Li and Ricke [124]
*E. coli* Δ*lysA* mini-Tn5-Km-*gfp*mut3	l – Lysine	Cell growth	OD; GFP fluorescence	Chalova *et al.* [113,114]
*E. coli* GUC 41 (*metC*^−^, *thr*^−^)	l – Methionine l – Threonine	β-galactosidase	β-galactosidase activity	Hitchins *et al.* [117]
E. coli MD 33 *trpEA2*^−^	l –Tryptophan	β-galactosidase	β-galactosidase activity	Hitchins *et al.* [117]

**Table 3.** Comparison between lysine assimilation by small intestine and *E. Coli*.

**Features**		**Small intestine**	***E. coli***
Amino Acid transport	Mechanism	Passive diffusion, facilitated diffusion, active transport Active: energized by Na^+^-membrane gradient	Passive diffusion, facilitated diffusion, active transport Active: energized by H^+^-membrane gradient (*lysP*) and ATP-hydrolysis (LAO system)
	Stereospecificity	Only l-amino acids are transported	Only l-amino acids are transported
Peptide Transport	Transporters	Distinct from amino acid transporters	Distinct from amino acid transporters
	Peptide size	Di- and three-peptides	Di- and three-peptides
	Stereospecificity	Peptides consist of l-amino acids	Peptides consist of l-amino acids
	N- and C-terminal groups	Required for transport	Required for transport
